# Health-related quality of life, mental health and caregiver burden in children with autosomal recessive polycystic kidney disease

**DOI:** 10.1007/s00467-025-06795-1

**Published:** 2025-09-18

**Authors:** Charlotte Gimpel, Susanne Schaefer, Franz Schaefer

**Affiliations:** 1 Division of Pediatric Nephrology, Center for Pediatrics and Adolescent Medicine, University Hospital Heidelberg, Heidelberg, Germany; 2 Praxis Für Kinderkardiologie Und Kindernephrologie, Medizinisches Versorgungszentrum Des Klinikum Konstanz, Constance, Germany

**Keywords:** Pediatric chronic kidney disease, Patient-reported outcome, Health-related quality of life, Caregiver burden, COVID pandemic

## Abstract

**Background:**

Pediatric chronic kidney disease (CKD) causes significantly impaired health-related quality of life (hrQOL) and caregiver burden, but no studies focus specifically on autosomal recessive polycystic kidney disease (ARPKD).

**Methods:**

This prospective case–control study assessed hrQOL (using PedsQL®ESRD) and screened for psychosocial problems (strength and difficulties questionnaire (SDQ)) in 43 children with ARPKD. Fifty-eight caregivers reported on the disease’s impact on family (FaBel) and their own QOL (Ulm inventory of parental caregiver QOL (ULQIE)). As controls, we questioned 36 matched healthy children and 57 parents under similar pandemic restrictions and used published historical controls (healthy and with advanced CKD).

**Results:**

Patients were aged 9.0 ± 4.8 years with CKD stage G1–4 (45%), on dialysis (14%) or after kidney transplantation (26%). Nine patients had developmental delay secondary to medical complications. PedsQL®ESRD total scores correlated significantly to kidney function, but could not capture liver-specific symptoms. All 4 measures showed significant differences between treatment modalities with best scores in patients during CKD stages G1–4 and worst on dialysis, except SDQ, which was worst after transplantation. The most significant extra-renal risk factor for all 4 scores was developmental delay of the child. SDQ scores were elevated in contemporary vs. historical controls, but even further in ARPKD especially for peer relationship problems.

**Conclusion:**

In summary, ARPKD causes significantly impaired hrQOL, psychosocial problems and caregiver burden, which were equal to, if not greater than, that of controls with more advanced kidney failure. Treatment modality and developmental delay were the most important risk factors.

**Trial registration:**

Trial registered 06/2020 DRKS S00021059.

**Graphical abstract:**

**Figure:**
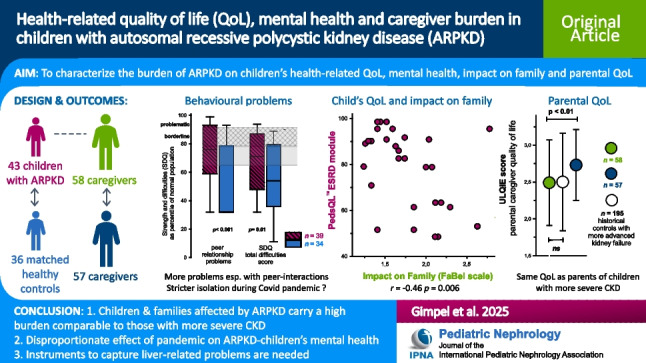
A higher resolution version of the Graphical abstract is available as Supplementary information

**Supplementary Information:**

The online version contains supplementary material available at 10.1007/s00467-025-06795-1.

## Introduction

Autosomal recessive polycystic kidney disease (ARPKD) is a rare but particularly severe and complex cause of childhood chronic kidney disease (CKD) which usually also affects the liver. About two-thirds of children present perinatally with many suffering from pulmonary hypoplasia secondary to intrauterine oligohydramnios and massively enlarged kidneys [1], while a smaller group presents later in childhood or even young adulthood with more predominant liver involvement [2]. Studies have documented the large psychological burden that children with all stages of CKD carry and the enormous strains on their families [3–9], but the subpopulation of ARPKD may represent a more severely affected cohort due to their predominantly early and severe presentation and multi-organ involvement. Even though there are specific instruments to capture the burden of symptoms and treatment of childhood kidney failure as well as the burden on parental caregivers,^1^there are no ARPKD-specific instruments. The first aim of this study was therefore to assess the burden of ARPKD in young patients with ARPKD and their families using several established instruments with available historical pediatric controls who were either healthy or had other chronic health conditions. Disease effect on children was captured both for everyday symptoms and health effects (health-related quality of life (hrQOL)) as well as emotional and behavioural problems. The effect of the disease on the family was conceptualized both in terms of the impact on family life (including daily living, siblings’ strains and financial burden), as well as the parental QOL including their physical well-being, self-fulfilment and satisfaction with family life. An exploratory aim was whether specific disease characteristics such as extent of abdominal distention or perinatal presentation made a measurable difference on these QOL indices. 

As research on polycystic kidney diseases has generated a number of potential novel treatments [10], there is a need to validate patient-reported outcome measures in this rare-disease population in order to adequately capture patient and family impact of these novel therapies in future clinical trials. The second aim of this study was therefore to validate existing instruments for use in ARPKD studies by describing statistical properties in this population, to assess criterion validity by demonstrating dependency on kidney function and treatment modality and to describe the short-comings of available generic instruments for use in ARPKD.

As the COVID pandemic coincided with the planned data-acquisition phase of the study, and the huge emotional and social impact of social restrictions on children and adolescents became evident [11–13], recruitment to the study was delayed until a control group of healthy children living under similar pandemic restrictions could be included in the study. It has now become clear from multiple longitudinal analyses that the profound effects have lasted well beyond the duration of the contact and schooling restrictions [14, 15]. Increased mental health problems and decreased hrQOL in otherwise healthy adolescents have been found in numerous studies [15]. However, less is known about the specific interaction of an ongoing chronic illness and stresses of the pandemic in children and adolescents. In Europe, it has mainly been examined without contemporary controls, e.g. for rheumatoid diseases [16], type 1 diabetes mellitus [17] and atopic diseases [18], with very little information on children with immunocompromising diseases compared to controls [19]. A large online study in Chinese children with CKD from mixed causes conducted very early in the pandemic (March 2020) demonstrated no difference in antisocial or neurotic behaviour compared to healthy children but higher self-assessed anxiety and depression in their guardians compared to parents of healthy children [20]. European data also supports the increased stress of parenting a child with an immunocompromising disease during the pandemic [21]. Therefore, a third aim of this study was to assess the impact of the pandemic restrictions on the mental health and hrQOL of children with ARPKD and to describe correlations to both the burden of the chronic disease on the family and the parental QOL.

## Methods

### Study design and instruments

For this cross-sectional case–control study, the following previously validated questionnaires with official German versions were chosen. Patients’ hrQOL was assessed using PedsQL® ESRD 3.0 module [22] (range 0–100, higher scores indicate higher QOL). For self-reports, age-appropriate wordings for 5–7-year-, 8–12-year-, 13–18-year- and 18–25-year-olds were administered, while for parental proxies content-adapted versions for 2–4-year-, 4–17-year- and over 18-year-old patients were used. The PedsQL® ESRD 3.0 module is a 34-item questionnaire with subscales on (1) Fatigue (4 items), (2) Renal symptoms (5 items), (3) Treatment problems (4 items), (4) Social interactions (3 items), (5) Worry (10 items), (6) Perceived physical appearance (3 items) and (7) Communication with healthcare professionals (5 items) [22]. Cronbach’s *α* for proxy-reported subscales ranges from 0.71 to 0.93 [22]. The Strengths and Difficulties Questionnaire (SDQ) was used as a brief emotional and behavioural screening tool. The 25 items were completed by at least one parental proxy [23] (with age-appropriate versions for 2–4-year-, 4–17-year- and over 18-year-olds), and self-reports by patients aged 11 and above [24, 25] (with age-appropriate versions for 11–17-year- and over 18-year-olds). It has 5 subscores for (1) Emotional symptoms, (2) Conduct problems, (3) Hyperactivity/inattention, (4) Peer relationship problems and the (5) Prosocial behaviour, with 5 items each. The total problem score is the mean of subscores 1 to 4, with higher scores indicating more problems. Psychometric properties have been extensively reported internationally [25], including in German children and parents with proxy-reported Cronbach *α* for the subscales between 0.58 and 0.76 [26, 27]. Caregiver and family burden were assessed by the parents with the validated German version of the Impact on Family Scale (FaBel) [28, 29], which has Likert scale items on the social and familial consequences of living with a child with a chronic health condition and has demonstrated good internal consistency and discriminant validity and acceptable construct validity [29]. We applied the scoring of the original German translation [29] (mean scores (range 1–4, with higher scores indicating higher impact) as well as the German 27-item total score and division of subscales, as factor analysis of the validation cohort demonstrated a slightly different structure to the original US population [29]. Caregiver QOL was assessed with the Ulm Quality of Life Inventory for Parents of Chronically Ill Children (ULQIE) which is a 29-item questionnaire on the dimensions of physical and daily functioning, satisfaction with the situation in the family, emotional distress, self-development and well-being with higher scores indicating higher QOL [30]. In German parents of children with malignant diseases, type 1 diabetes and epilepsy it demonstrated good consistency and test–retest reliability as well as discriminant validity between acute and chronic phases of coping with a novel diagnosis [30]. The parents additionally answered a demographic questionnaire about their child and themselves which included medical questions such as whether the disease had been genetically confirmed, current height, weight, serum creatinine, abdominal circumference, timing of any kidney replacement therapy and other life-changing diseases. Questions on social background included parental ethnic background, number and age of siblings, type of childcare/school attended, parental occupation (hours per week) and who was the main caregiver regarding usual childcare and medical care.

Healthy children as controls and their caregivers completed questionnaires at similar time points during the pandemic restrictions which included only those questions which could be meaningfully posed to healthy controls: i.e. the full SDQ and ULQIE questionnaires and an abbreviated PedsQL®ESRD (questions on fatigue, renal symptoms (headache, dizziness, edema, thirst and muscle cramps), difficulties to drink/eat what is expected). Additionally, all caregivers were asked to rank on a 5-point Likert scale whether/how they perceived the impact of the pandemic on their answers (very negative, negative, only slight, no difference, positive impact on QOL). Historical controls were taken from large populations of healthy children tested prior to the pandemic as follows. For the SDQ, scores were expressed as percentiles of normal values based on the publications of Woerner et al. for proxy scores [26] and Becker et al. for self-assessment scores [27]. ULQIE score percentiles are based on a reference population of parents of children with other chronic diseases (cancer, diabetes and epilepsy) [30], but were also compared to a German cohort of parents of children with advanced chronic kidney failure for whom only mean and SD values were available [3]. Children in the latter study by Wiedebusch et al. had often received a kidney transplantation (62%), with 16% on dialysis and 16% CKD stage G1–4 and 6% unknown treatment modality [3].

### Setting and participants

All children and young adults with a clinical or genetically confirmed diagnosis of ARPKD from the age of 5 were eligible for the study, as well as parents and permanent caregivers of 0–25-year-old ARPKD patients who were themselves over the age of 18 years (including grandparents or stepparents, if they live in the same household). The only exclusion criteria were insufficient understanding of the questionnaires due to intellectual capabilities or language barriers and lack of written informed consent. Families were recruited from German pediatric nephrology clinics by an invitation letter via their treating physician and via personal invitation at an ARPKD patients’ day as well as invitations on social media websites of patient associations. Details about duration and extent of kidney disease, number of medications and developmental delay were provided directly by the proxies. Only the abdominal circumference was measured at time of interview.

Control families were eligible if the child did not have a life-changing disease; parental chronic illness was not an exclusion criterion, as this was not an exclusion criterion in affected families either. Controls were recruited via personal contacts of CG or of families who either took part in the study or in an ARPKD patients’ day.

Questionnaires were administered to affected families on paper after prior explanation by SS or CG, mostly in the family homes, but also via video call (*n* = 5) and in a private side-room at a patients’ information day (*n* = 5) between 21/07/2020 and 24/09/2022 (median 04/06/2021). Controls answered questionnaires on paper in their own homes between 05/07/2021 and 02/12/2022 (median 30/09/2021). The study was approved by the institutional review boards of the Universities of Freiburg (reference 162/20) and Heidelberg (reference S-792/2020), Germany, and registered in the German Register of Clinical Trials (DRKS S00021059). Written informed consent was obtained for all participants. Relevant licenses/author permissions were secured for all instruments (see ‘Acknowledgements’).

### Statistics

Answers were manually transferred into Excel^®^ by SS and CG and analyzed using SAS^®^ v9.4. If two proxies had answered for a given patient, the mean score was taken for each answer and the sub- and total scores calculated consequently. Distribution was visualized for all scores with manual checks for ceiling effects. Psychometric properties of the instruments in the cohort of affected children were examined within the framework of classical test theory; this supposes that all observed scores are composed of a true score and a random error score and that the reliability of a scale (in terms of all sub-questions measuring the same concept) can be assessed with Cronbach’s α (or tau-equivalent reliability (ρτ)) which is based on correlation of the individual items. We adopted the generally agreed value of Cronbach’s alpha above 0.7 to reflect adequate internal consistency of the scales [31]. Criterion validity of the symptom scales was tested by correlation to eGFR, treatment modality and abdominal distention. The eGFR was estimated with the Schwartz 2009 bedside formula; it was set to 5 ml/min*1.73 m^2^ if it was unknown in a patient on dialysis and to 140 ml/min*1.73 m^2^ in 2 patients with hyperfiltration. Standard deviation scores for abdominal circumference were calculated with the LMS method based on data published from Frederiks et al. [32].

As most of the SDQ, PedsQL^®^ESRD, ULQIE and FaBel subscales and total scales did not show normal distribution (only 7 of 46 were compatible with normal distribution on the Shapiro–Wilk testing), we used non-parametric tests for group comparisons, i.e. the Wilcoxon test for comparison of continuous variables between two groups and the Kruskal–Wallis test for more than two groups (e.g. treatment modality). Frequencies of categorical variables were assessed with chi square test. For the comparison to the previously published historical controls with advanced chronic kidney failure [3], a non-parametric comparison was not possible, because the patient-level data are no longer available (personal communication). Therefore, Welch’s *t*-test was used which assumes normal distribution, but in contrast to Student’s *t*-test does not assume equal variance. Correlations are given as Pearson’s correlation coefficients. *p* values < 0.05 were considered significant.

## Results

### Demographics of affected children

Of approximately 60 families approached, 41 families affected by ARPKD agreed to take part in the study. Most families who refused did so because of time constraints. We know of 4 families that were not approached by the treating physician due to known language barrier. Two family units were excluded because they consisted only of a young adult over 25 years with ARPKD. For 15 children, only the parents participated as proxies and caregivers, but the children were not questioned directly even though they were old enough, either because the parents did not want their children to focus on possible symptoms (*n* = 5), because of developmental delay (*n* = 6) or because the child did not want to take part (*n* = 4). More detailed information on how many eligible patients, proxies and caregivers completed the questionnaires is given in Table S1.

In the final analysis, we included 43 children with ARPKD from 39 different families and 61 caregivers, as well as 36 healthy control children from 35 different families with 57 of their caregivers. Demographic details of patients and controls are given in Table 1a. There were four pairs of siblings in the ARPKD group and one in the control group.

**Table 1:** Characteristics of patients, healthy control children and caregivers

	ARPKD	Healthy controls	*p*
Family units, *n* =	39	35	
**a. Child characteristics**			
Children/young persons, *n* =	43	36	
Age [years]	9.0 ± 4.8	8.8 ± 5.0	ns
Female, *n* =	20 (47%)	19 (53%)	ns
No of caregivers for this child	1.9 ± 0.4	1.9 ± 0.4	ns
No of siblings	1.1 ± 0.8	1.1 ± 0.8	ns
German mother, *n* =	31 (72%)	29 (81%)	ns
German father, *n* =	32 (74%)	25 (69%)	ns
Height [cm]	127.6 ± 26	135.1 ± 29	ns
Height SDS [*z*-score]	− 1.14 ± 1.35	0.31 ± 1.09	< 0.0001
**b. Caregiver characteristics**			
Caregivers, *n* =	58	57	
Mothers, *n* =	36 (62%)	34 (60%)	ns
Age of caregiver [years]	41 ± 6.3	42 ± 6.7	ns
Single parent, *n* =	2 (3%)	5 (9%)	ns
N^o^ of dependent children	1.8 ± 0.9	2 ± 0.8	ns
Stay-at-home parent, *n* =	13 (22%)	6 (11%)	ns
Paid work per week [hours]	24 ± 16	27 ± 13	ns
Mothers who said they take on:			
> 50% medical care	29/36 (81%)	28/29 (97%)	ns (0.10)
> 50% childcare	29/36 (81%)	28/31 (90%)	ns (0.09)
Fathers who said they take on:			
> 50% medical care	2/22 (9%)	2/20 (10%)	ns
> 50% childcare	2/22 (9%)	4/21 (19%)	ns
Family units, *n* =	39	35	
Unique child-caregiver pairs	63	58	

*ARPKD*, autosomal dominant polycystic kidney disease; *SDS*, standard deviation score; *ns*, not significant

*p* values are for the Wilcoxon test/chi-square test comparing means/proportions between affected and control groups

As detailed in Table 2, affected children represented the whole spectrum of ARPKD in childhood from birth to age of majority, from classical severe neonatal onset to diagnosis at age 11 with liver-predominant phenotype and from preserved kidney function to combined liver/kidney transplantation. Four families had two affected children and another two families had lost a previous child to ARPKD. Disease characteristics for affected ARPKD children in each of the questionnaire subgroups are given in Table S2.

**Table 2:** Disease characteristics in 43 ARPKD patients

	**Non-missing**	**Mean ± SD**	**Range**
Age at diagnosis [years]	43/43	1.1 ± 2.7	- − 0.47^a^ to 11
Age at interview [years]	43/43	9.0 ± 4.8	0.5–21.4
eGFR [ml/min*1.73 m^2^]	33/39	71 ± 47	4–140
Time on current KRT [years]	12/18	3.1 ± 2.2	0–6.6
N^o^ of different drugs taken	41/43	5.2 ± 4.2	0–16
Abdominal circumference [cm]	19/31^b^	66 ± 14	46–101
Abdominal circumference SDS	19/31^b^	1.5 ± 1.7	− 0.5 to 5.6
	**Non-missing**	***n***	**% of respondents**
CKD stage G1–4	42/43	19	45%
CKD stage G5D on PD or HD	42/43	4, 2^c^	10%, 5%
Functioning graft after kidney, or liver and kidney transplant	42/43	9, 2	21%, 5%
Nephrectomy: both, one, none	43/43	10, 2, 31	23%, 5%, 72%
Perinatal presentation	43/43	31	72%
Developmental delay	43/43	9	21%

*SD*, standard deviation*; KRT*, kidney replacement therapy*; SDS*, standard deviation score*; PD*, peritoneal dialysis; *HD*, haemodialysis

^a^Negative age denotes diagnosis before birth (also in years)

^b^Only patients who had not undergone nephrectomy

^c^Of which 1 had a non-functioning kidney graft

Nine children had developmental delay which was a direct or indirect complication of ARPKD in all cases (e.g. hypoxic-ischemic encephalopathy after premature birth with pulmonary hypoplasia or abdominal dystocia, hypoxic and ischemic stroke after anaesthesia for PEG placement). Of these, one 12-year-old girl completed the PedsQL^®^ESRD questionnaire for 5–7-year-old children, 6 did not complete any self-assessments and 2 were not eligible for self-assessment due to their age.

### Health-related QOL in affected children

The mean proxy-reported PedsQL^®^ESRD total score was 78.1 ± 10.6 (range 59–96). It showed no ceiling or floor effect and was slightly lower than the self-reported total score of 79.2 ± 16.6 (range 49–99). Internal consistency of the self- and proxy-reported total scales was good (Cronbach’s α 0.95 and 0.88), but quite variable in the subscales (see Table S3). The PedsQL^®^ESRD subscores for fatigue and renal symptoms, as well as difficulties regarding the recommended intake of fluid and dietary restrictions, differed significantly between children with ARPKD and healthy controls (see Table S3).

### Mental health in affected children

**Fig. 1:**
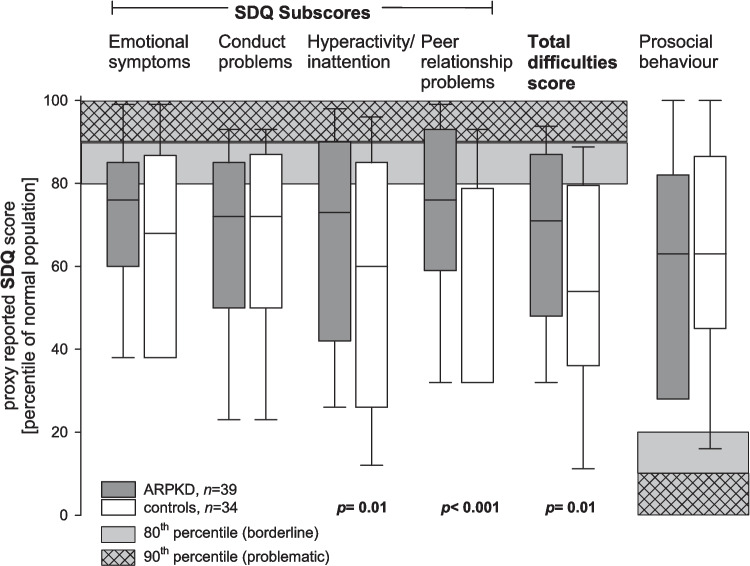
Strength and difficulties questionnaire (SDQ): proxy-reported subscores and total score in children with ARPKD and healthy control children expressed as percentiles of normal, pre-COVID population [26]. Box: 25th, 50th and 75th percentile. Whiskers: 5th and 95th percentile. *p* values are for one-sided Wilcoxon test of raw SDQ scores of ARPKD vs. contemporary controls. Percentile of normal population taken from [26]. ARPKD, autosomal recessive polycystic kidney disease

Full results of the SDQ behavioural screening questionnaire are given in Table S4. The internal consistency of the total SDQ was good for the proxy-rated (*α* = 0.79) and fair for the self-rated total scores (*α* = 0.69). The subscores performed mainly adequately when proxy-rated but often inadequately when self-rated (see Table S4). Higher scores in the group of children with ARPKD (compared to current and historical healthy controls) only led to slight ceiling effects (see Fig. 1). Specifically, the proxy-rated total difficulties score, peer relationship problems and hyperactivity/inattention subscores were significantly higher in children with ARPKD than in healthy controls (see Table S4 for absolute values and Fig. 1 for scores expressed as percentiles of a pre-COVID normal population). Notably, a higher rate of contemporary healthy control children also reported borderline or abnormal values than the pre-COVID healthy children (Fig. 1). Children affected by ARPKD had most pronounced problems with peer relationships, while this was the least affected subscale among healthy controls (Fig. 1). Affected children rated their own problems lower than their parental proxies, while healthy children rated them higher than their proxies had reported (Table S4).

## Caregiver burden–demographics of caregivers

Questionnaires on caregiver burden were completed by 61 parents (61% mothers) of children with ARPKD and 57 parents (60% mothers) of healthy control children. All proxies were biological parents, except for two step-fathers of affected children and one mother who had adopted a healthy toddler. No grandparents or non-parental caregivers took part. Demographic details of caregivers are given in Table 1b and Table S5.

### Caregiver quality of life

**Fig. 2:**
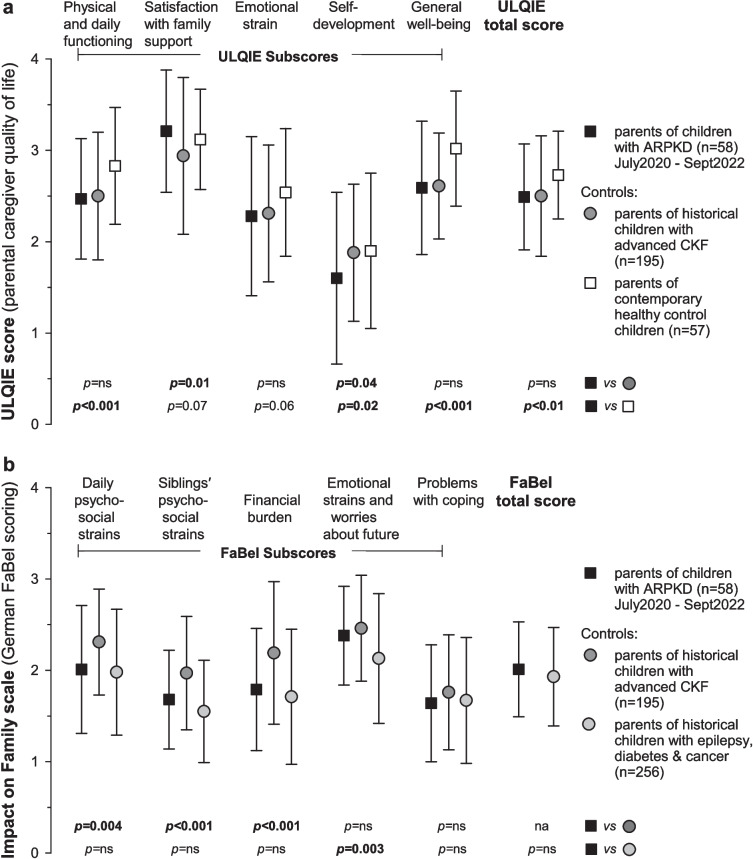
**a** Ulm inventory for parental quality of life (ULQIE) in parents of children with ARPKD vs. historical controls affected by advanced CKF taken from reference [3] vs. contemporary healthy controls. **b** Impact on family scale (FaBel) in parents of children with ARPKD vs. historical controls affected either by advanced CKF [3] or epilepsy, diabetes or cancer [29] (mean and standard deviations). Data collected during pandemic restrictions in squares. Historical data (before the COVID pandemic) in circles. Data from healthy children in white. ARPKD, autosomal recessive polycystic kidney disease; CKF, chronic kidney failure. *p* values comparing ARPKD vs. historical CKF controls [3] are for Welch’s *t*-test; *p* values comparing ARPKD vs. contemporary healthy controls and historical controls with epilepsy/diabetes/cancer [29] are for Wilcoxon’s test

ULQIE total and subscales consistently showed good internal consistency in parents of both affected and healthy children (see Table S6). There were only slight ceiling effects for parents of healthy children in the subdomains of physical functioning and satisfaction with family support. Parents of children with ARPKD reported significantly lower scores for their own QOL than the contemporary healthy controls for the ULQIE total score, physical and daily functioning and general well-being (see Fig. 2a and Table S6). ULQIE scores were generally similar between parents of children with ARPKD compared to pre-pandemic families of children with advanced chronic kidney failure (CKF) (see Fig. 2a). Among parents of children with ARPKD, there was no significant difference in ULQIE total score between mothers and fathers, and those who took on more or less than 50% of the child’s medical care. Also, there was no correlation to age of the parent, hours of paid work per week or number of other dependent children of that parent.

### Impact on family life (FaBel)

The FaBel 27-item total scores covered nearly the entire possible range with no ceiling effect (mean 2.01 ± 0.52, range 1.44–3.44 (possible range 1–4)). Internal consistency of the total score, as well as the abbreviated 11-item total score, was very good, but more variable in the subscales (see Table S6). The FaBel total score was not rated differently between mothers and fathers, or those who took on more or less than 50% of the child’s medical care. Also, there was no correlation of FaBel total score to age of the parent, hours of paid work per week or number of other dependent children of that parent.

## Association between different scales

### Within children–hrQOL and psychosocial problems

The PedsQL^®^ESRD total score was most closely related to the SDQ emotional problems subscore both as judged by proxies (*r* =  − 0.66, *p* < 0.001, *n* = 33) as well as affected children themselves (*r* =  − 0.78, *p* < 0.001, *n* = 16). The SDQ total score was most closely associated with the PedsQL^®^ESRD subscore for communication as judged both by proxies (*r* =  − 0.51, *p* = 0.002, *n* = 34) and patients (*r* =  − 0.51, *p* = 0.04, *n* = 27). The two most significantly associated subscores were SDQ emotional problems and PedsQL^®^ESRD fatigue (proxies: *r* =  − 0.44, *p* = 0.005, *n* = 38; patients: *r* =  − 0.75, *p* = 0.0008, *n* = 16). These correlations existed despite only very limited overlap of item contents.

### Within caregivers–quality of life and impact on family

There were significant strong correlations between ULQIE (total and all subscores except satisfaction with family support), with the impact on family scale (total scores and all subscores except coping) (see Table S7) in the group of 57 caregivers of affected children who had answered both scales. The correlation between the total ULQIE and total FaBel score was high with* r* = 0.72 and *p* < 0.0001.

### Associations of caregiver burden with affected child’s problems

**Fig. 3:**
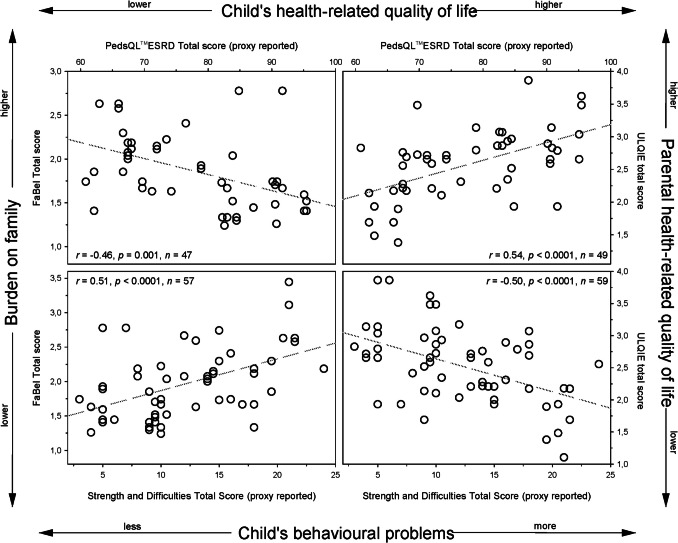
Correlation of health-related quality of life and behavioural problems in children with ARPKD with impact on family life and parental quality of life. ARPKD, autosomal recessive polycystic kidney disease; FaBel, impact on family scale (with German FaBel scoring system); ULQIE, Ulm quality of life inventory for parents of children with a chronic illness; *r*: Pearson’s correlation coefficient; *n*: number of unique parent–child pairs

Among the unique pairs of affected child and caregiver, there were significant correlations of both child’s hrQOL and SDQ score with both impact on family and parental QOL (see Fig. 3). The strength of these correlations was even slightly stronger for the self-reported child indices: PedsQL^®^ESRD with FaBel *r* =  − 0.49, *p* = 0.006, *n* = 30, PedsQL^®^ESRD with ULQIE *r* = 0.69, *p* < 0.0001, *n* = 31, SDQ with FaBel *r* = 0.49, *p* = 0.017, *n* = 23, SDQ with ULQIE *r* =  − 0.54, *p* = 0.007, *n* = 24.

## Impact of kidney function and treatment modality

**Fig. 4:**
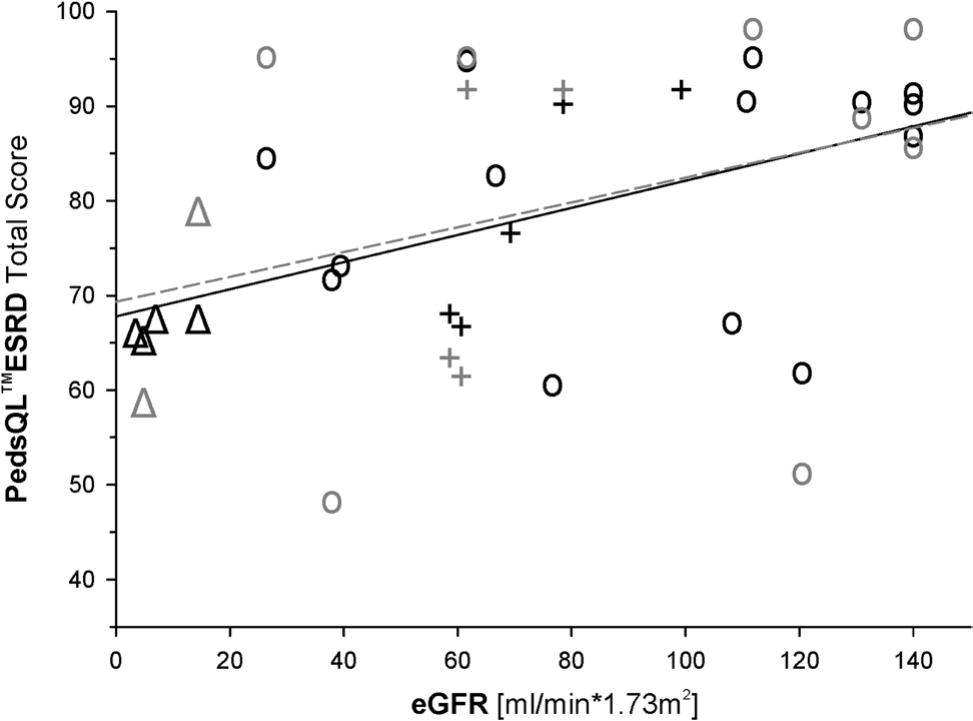
Scatter plot of eGFR with PedsQL^®^ESRD total scores (proxy- and self-reported) with regression lines and Pearson’s correlation coefficients. O CKD stage G1–4, Δ dialysis, + functioning graft; proxy-reported: black symbols (*n* = 23, *r* = 0.54, *p* < 0.01), solid line (Total Score = 67.8 + 0.143*eGFR); self-reported: grey symbols (*n* = 14, *r* = 0.33, *p* = ns), dashed line (Total Score = 69.4 + 0.131*eGFR)

Kidney function and treatment modality (CKD stage G1–4, dialysis or transplantation) had significant impact on nearly all scores examined. As detailed in Table 3, there was a significant difference in PedsQL^®^ESRD total scores with highest (best) scores during CKD stage G1–4, worst on dialysis and intermediate scores if the children had a functioning kidney graft. The relationship between eGFR and PedsQL^®^ESRD scores was very similar for both children (self-report) and parents (proxy-report) (see Fig. 4). In contrast to the association with kidney function, there was no significant correlation of PedsQL^®^ESRD total or subscores with abdominal circumference (absolute or SDS) in patients who had not undergone nephrectomy (*n* = 19).

**Table 3:** Child and parental quality of life indices of children with ARPKD by treatment modality

	CKD stage G1–4				Dialysis					Functioning kidney graft			
Variable	*n*	Mean	SD	Range	*n*	Mean	SD	Range	*n*	Mean	SD	Range	*p*
PedsQL®ESRD Total proxy-rated	21	81.1	10.3	(60.8–95.5)	4	66.5	1.13	(65.2–67.4)	8	76.0	10.0	(63.0–91.7)	**0.03**
PedsQL®ESRD Total self-reported	12	83.6	17.3	(48.5–98.5)	2	68.7	14.2	(58.6–78.8)	7	74.7	15.5	(53.0–91.7)	ns
SDQ Total Score proxy-rated	24	9.54	5.34	(3–24)	5	13.80	5.32	(6.5–21.5)	10	15.30	4.84	(7–21)	**0.002**
SDQ Total Score self-reported	7	7.57	2.94	(5–13)	2	11.50	2.12	(10–13)	8	13.00	6.21	(6–23)	ns
ULQIE Total Score	25	2.74	0.49	(1.9–3.8)	8	2.14	0.56	(1–2.8)	16	2.20	0.64	(1.1–3.5)	**0.01**
FaBel Total Score	25	1.78	0.38	(1.3–2.8)	8	2.42	0.42	(2–3.2)	16	2.30	0.58	(1.2–3.4)	**0.005**
Age at interview [years]	25	7.85	3.75	(0.50–14.3)	7	9.18	7.37	(1.1–21.4)	11	11.48	4.70	(3.7–19.3)	ns
Age at diagnosis [years]	25	1.59	2.77	(− 0.24 to 11)	7	1.42	4.22	(− 0.3 to 11)	11	− 0.11	0.15	(− 0.5 to 0)	**0.018**
eGFR [ml/min*1.73 m^2^]	18	92	41	(25–140)	7	6	4	(3.6–14.6)	8	82	24	(59–118)	**0.0003**
Height SDS [z-score]	25	− 0.84	1.14	(− 2.88 to 1.43)	6	− 1.69	0.63	(− 2.3 to 0.8)	11	− 1.51	1.90	(− 4.8 to 1.7)	**0.004**
N^o^ of different medications taken	25	3.68	2.93	(0–10)	5	8.80	5.76	(0–16)	11	7.18	4.40	(0–15)	** < 0.0001**
Perinatal presentation		14	of 25	(= 56%)		6	of 7	(= 86%)		11	of 11	(= 100%)	**0.02**
Developmental delay		4	of 25	(= 16%)		1	of 7	(= 14%)		4	of 11	(= 36%)	ns

*PedsQL®ESRD*, pediatric quality of life inventory – end stage kidney disease module (for children from 5 years and proxies of children from 2 years of age); *SDQ*, strength and difficulties questionnaire (for children from 11 years and proxies of children from 2 years of age) (if both parents answered mean was taken); *ULQIE*, Ulm quality of life inventory for parents of a child with chronic illness (if both parents answered mean was taken); *FaBel*, impact of family scale for families with an affected child; *ns*, not significant

*p* values are for the non-parametric Kruskal–Wallis test comparing all 3 groups

There was also a significant difference between the treatment modality groups for SDQ scores (see Table 3), but in contrast to the PedsQL^®^ESRD, children with functioning grafts did not fare better than those on dialysis but deteriorated further. Consequently, there was no correlation of SDQ total scores with eGFR.

Parental QOL (total ULQIE score) was also correlated to child’s eGFR (*r* = 0.46, *p* = 0.009) and number of different medications prescribed (*r* =  − 0.47, *p* = 0.003), but not to child’s age, height or abdominal distention. Compared to a historical cohort of parents of children with CKF (which had a mean ULQIE score of 2.50 ± 0.66, *n* = 195), the group of parents of children with ARPKD had a very similar mean ULQIE score of 2.49 ± 0.58 (*n* = 58) [3] (for subscales see Fig. 2a). However, in the historical control group, there were no children with good kidney function, and when only children on dialysis and those living with functional kidney grafts were compared, mean parental ULQIE scores were lower in the ARPKD group (see Table S8, no significance testing possible, as SD values no longer available in historical control group).

The disease’s impact on family as measured by FaBel total scores correlated with the eGFR (*r* =  − 0.49, *p* = 0.005) with significant differences between children on dialysis vs. with functioning graft vs. CKD stage G1–4 (*p* < 0.001, see Table 3). FaBel scores were also correlated to total number of prescribed medicines (*r* = 0.59, *p* < 0.0001) and amount of abdominal distention (*r* = 0.40, *p* = 0.037). The five FaBel subscales were compared to a historical control population of families with children with all-cause CKF [3], and were generally slightly lower for families affected by ARPKD (see Fig. 2b and Table S9). Compared to a historical cohort of parents of children with other chronic illnesses (epilepsy, diabetes and cancer) [29], parents of children with ARPKD had significantly more worries about the future (*p* < 0.003, see Fig. 2b) and similar scores on the other subscores and total score.

### Impact of presentation of ARPKD

The group of children who had initially presented perinatally did not have lower hrQOL or self-reported mental health problems than those who presented later in life (see Table S10). However, parental QOL was lower and the impact on the family higher in children with perinatal presentations (see Table S10). Consequently, age at presentation was correlated to proxy-rated SDQ (*r* =  − 0.38, *p* = 0.017), ULQIE total score (*r* = 0.45, *p* = 0.003) and FaBel total score (*r* =  − 0.37, *p* = 0.018).

### Impact of developmental delay

Table 4 illustrates that children who suffered developmental delay due to a complication of ARPKD had significantly lower hrQOL and more psychosocial problems (see Fig. S1 and Fig. S2 for details of SDQ subscores). Also, their parents reported lower scores on their own QOL and a higher impact on family.

**Table 4:** Child and parental quality of life indices of children with ARPKD and with vs. without developmental delay (due to complications of ARPKD)

	Without developmental delay				With developmental delay					
Variable	*n*	Mean	SD	Range	*n*	Mean	SD	Range	***p***	
PedsQL^®^ESRD Total, proxy rated	27	80.0	10.8	(61–95)	6	69.6	5.1	(63–77)	**0.02**	
SDQ Total Score, proxy rated	30	10.1	4.92	(3–19.5)	9	16.6	5.6	(8–24)	**0.003**	
ULQIE Total Score	32	2.65	0.59	(1.03–3.86)	9	2.15	0.37	(1.64–2.68)	**0.005**	
FaBel Total Score	32	1.88	0.49	(1.24–3.22)	9	2.40	0.45	(1.63–3.28)	**0.004**	
Age at interview [years]	34	9.5	5.1	(1–21)	9	7.1	3.1	(3–12)	**0.09**	
Age at diagnosis [years]	34	1.37	3.00	(−0.5–11)	9	0.24	1.04	(−0.24–3)	0.12	
eGFR [ml/min*1.73 m^2^]	26	70	49	(5–140)	7	79	43	(4–118)	ns	
Height SDS [*z*-score]	33	−0.98	1.19	(−3–2)	9	−1.73	1.78	(−4.77–1.17)	ns	
Nr of different medications taken	33	5.2	4.1	(0–16)	8	5.6	4.9	(0–15)	ns	
Perinatal presentation		23	of 34	(= 68%)		8	of 9	(= 89%)	ns	
Treatment modality CKD stage G1–4		21	of 34	(= 62%)		4	of 9	(= 44%)	ns	

*PedsQL®ESRD*, pediatric quality of life inventory – end stage kidney disease module (for children from 5 years and proxies of children from 2 years of age); *SDQ*, strength and difficulties questionnaire (for children from 11 years and proxies of children from 2 years of age); *ULQIE*, Ulm quality of life inventory for parents of a child with chronic illness (if both parents answered mean was taken); *FaBel*, impact of family scale for families with an affected child (if both parents answered mean was taken); *ns*, not significant

*p* values are for the non-parametric one-sided Wilcoxon test

PedsQL^®^ESRD and SDQ self-reports not included as only 1 child with developmental delay was able to answer this

### Multiple regression for impact of age at presentation, treatment modality and developmental delay

As there was a significant association between perinatal presentation with treatment modality (see Tables 3 and 5, e.g. no children with late presentation had required a transplant) and a non-significant trend for the association of perinatal presentation with developmental delay, the influence of all three on our measures was examined in multiple linear regression models. These revealed that perinatal presentation was not a significant independent predictor of child’s symptoms or caregiver burden, but developmental delay had independent predictive value (see Table 5). For the SDQ, developmental delay was the only independent predictor, while treatment modality and perinatal presentation were not independently correlated to total SDQ score.

**Table 5:** Multiple regression models to compare the impact of treatment modality (CKD stage G1–4 vs. dialysis vs. functioning graft), presentation (perinatal vs. later) and developmental delay (yes vs. no) on the total scores of the instruments

	*n*	r^2^	p for total model	Type III SS *p* values		
Dependent variable				Treatment modality	Presentation	Developmental delay
PedsQL®ESRD, proxy rated	33	0.328	**0.02**	**0.030**	0.336	**0.033**
SDQ, proxy rated	39	0.380	**0.002**	0.106	0.414	**0.009**
ULQIE	63	0.295	**0.0004**	**0.023**	0.460	**0.015**
FaBel	61	0.444	** < 0.0001**	**0.003**	0.370	**0.0004**

Type III SS *p* values (considering the impact of the other variables) for generalized linear models predicting dependent variable total scores

#### Perceived impact of the pandemic

When ranking the influence of the pandemic restrictions on their answers on quality of life, most families (57/76 = 75%) perceived no or only a small negative effect; however, it was more common for control families to report a negative or very negative impact (12/36 = 33%) than for families affected by ARPKD (7/40 = 18%, *p* = 0.11). More details are given in Fig. S3.

In contemporary healthy controls, the perceived impact of the pandemic was correlated to proxy-reported SDQ (*r* =  − 0.46, *p* = 0.006) as well as parental health (ULQIE *r* = 0.39, *p* = 0.003). However, in families affected by ARPKD, the perceived impact of the pandemic was neither related to PedsQL^®^ESRD total scores, ULQIE or FaBel scores and only correlated to self-reported SDQ (*r* =  − 0.62, *p* = 0.01).

## Discussion

This study presents the first disease-specific data on the impact of ARPKD on pediatric patients’ health-related quality of life (hrQOL), mental health and caregiver burden. We chose the PedsQL^®^ESRD instrument to capture hrQOL, the SDQ questionnaire to screen for emotional and behavioural problems, the ULQIE to assess parental QOL and the FaBel score to quantify the impact of the disease on the entire family because they have all been used in the same cultural context before and enabled comparison to historical pediatric controls who were either healthy or affected by other chronic kidney diseases. In terms of psychometric properties in the ARPKD population, the internal consistency, as measured by Cronbach’s *α*, was very good for the self-reported PedsQL^®^ESRD, ULQIE and FaBel scores. The PedsQL^®^ESRD proxy score was slightly less consistent, but still good, confirming previous reports of the local-language PedsQL^®^ESRD versions from Belgium [33], Brazil [34] and Malaysia [35]. The consistencies of ULQIE and FaBel in this study were also similar to those found in the initial studies [29, 30]. The SDQ score achieved only a good Cronbach *α* for the proxy version and a fair value in the self-reported version; however, this is consistent with its properties in large samples of adolescents from Germany and around the world [25, 27], suggesting that consistent concepts are captured in this subpopulation compared to the originally intended ones. The significant differences between SDQ and ULQIE scores in affected families versus contemporary healthy controls (see Fig. 1 and Fig. 2a) support their discriminant validity in reflecting the mental burden of living with and caring for a child with ARPKD. Regarding content validity and face validity, the kidney disease–specific PedsQL^®^ESRD questionnaire performed well in reflecting severity of kidney disease (see Fig. 4). However, the absence of a correlation between PedsQL^®^ESRD score and abdominal circumference (or its *z*-score) reflects the fact that PedsQL^®^ESRD is not designed to cover symptoms of liver enlargement and dysfunction, such as abdominal discomfort, bloating, poor appetite, gastrointestinal bleeding or itching, which are a special feature of ARPKD compared to other causes of childhood CKD. Unfortunately, there are no instruments available yet for capturing symptoms of children with portal hypertension. In our view therefore, the development of an ARPKD-specific patient-reported outcome measure to capture symptoms is needed, if future clinical trials in ARPKD are to reflect patient burden adequately.

In terms of documenting the burden of ARPKD on children, the hrQOL PedsQL^®^ESRD total scores of children on dialysis and after kidney transplantation were in a similar range to those previously reported for children with other causes of kidney failure [4]. The better PedsQL^®^ESRD scores post-transplant compared to on dialysis found here (see Table 3) confirmed a number of previous studies using self-reporting (see [4] for a meta-analysis), and proxy reports [7]. Fewer studies have reported PedsQL^®^ESRD in children with CKD prior to kidney failure, but those found here were higher than in children with CKD from Brazil and Thailand [36, 37]. The FaBel score indicated that the impact of ARPKD on family life was at a similar level as that of other chronic pediatric diseases (epilepsy, diabetes and childhood cancer) in the past [29]. While some aspects of caregiver burden caused by a child’s chronic disease are probably similar between these diseases (such as giving regular medications, supervision restrictions in diet, time spent for medical appointments), the greater scores of ARPKD parents in the subdomain of worries about the future may be either a pandemic effect or reflect the fact that the future necessity of a kidney transplant weighs heavy on parents even at an early stage of impaired kidney function [6]. Both impact on family (FaBel) and parental quality of life (ULQIE) total scores were similar to a group of parents of children with more severe kidney disease from 2010 [3], suggesting a more severe impact of ARPKD compared to other types of kidney failure. The emotional and behavioural screening questionnaire SDQ revealed a significantly increased total difficulties score compared to contemporary healthy controls (Fig. 1). This is consistent with previous reports in children with mixed cause CKD without controls which reported mental health difficulties using the SDQ [5, 38, 39] or other assessment tools [36]. However, a study with a large national control sample did not find a higher prevalence of a formal diagnosis of depression, anxiety or attention-deficit and hyperactivity disorder (ADHD) in children with CKD [40]. However, in that study, there was a poor correlation of a screening questionnaire on depressive symptoms and a formal diagnosis of depression. We speculate that the more ‘tangible’ effects of CKD distract healthcare professionals from mental and behavioural problems. The high correlation of total PedsQL^®^ESRD score and total SDQ score in our group should alert the clinician to be aware of the increased risk of psychosocial problems in children with more pronounced symptoms of their kidney disease. We found a high correlation of the child’s disease severity on parental stress levels and parental well-being, that is in line with recent studies using other instruments [8, 9, 36]. Perinatal presentation of ARPKD was a particular risk factor for higher caregiver burden (ULQIE and FaBel), but less for the child’s current hrQOL or emotional well-being. Overall, we could demonstrate that children with ARPKD carry a similar if not greater burden of kidney disease–related reduction in QOL and associated psychological problems, as well as burden on family life and parental reduction in QOL, than families affected by other chronic kidney diseases.

The most significant extra-renal risk factor for reduced hrQOL, emotional problems and higher caregiver burden was a co-existing developmental delay of the child. In multivariate analysis, it remained significant after adjusting for treatment modality. Interestingly, in the large Korean KNOW-Ped cohort study, both developmental delay and multi-organ involvement were also significant risk factors for mental health problems and adjustment problems [41] in children with CKD. While developmental delay is as an important factor for parental stress in both pre-school and school-age children [42, 43], it is also known to aggravate other parenting stressors such as poverty, history of immigration [42] or pandemic restrictions [44] and appears to be mediated by a lack of interactive engagement behaviours of the child [45]. As developmental delay was never due to an additional disease, but always secondary to a complication of ARPKD such as prematurity or severe hypotensive episodes, we emphasize the need for high-quality medical care for these children in order to avoid complications of ARPKD.

Our study opened the unplanned opportunity to study the interaction of the stress for children and their families of coping with CKD in addition to pandemic restrictions. It was notable that our healthy contemporary controls had higher SDQ problem scores than historical controls, especially for emotional symptoms, conduct problems and hyperactivity/inattention, which was most likely attributable to the pandemic. A large longitudinal population-based study in German children also showed a significant increase in the mean SDQ from pre-pandemic levels during 2020 and 2021 [12, 46], which only slightly decreased in autumn 2021 and 2022 [47]. This was true for all subdomains of the SDQ but most pronounced for hyperactivity symptoms, followed closely by conduct and peer problems and still noticeable for emotional problems [12]. Notably, in that cohort, SDQ scores recovered more slowly than a hrQOL measure as the pandemic progressed [47]. A study from Brazil in adolescents with a wider range of immunocompromising chronic diseases also showed increased SDQ during the pandemic, but not over and above that of contemporary healthy controls [19]. However, in our German ARPKD cohort, peer relationship problems were significantly increased above those of contemporary controls, which may reflect the fact that children with ARPKD were more strictly shielded from social contacts than healthy children during the pandemic, resulting in greater social isolation. A longitudinal study of children with nephrotic syndrome suggested that during the pandemic levels of fatigue rose [48]. However, from our data, it seems that fatigue was not increased more than in contemporary controls, especially in self-report (see Table S3). Despite the fact that parental quality of life (ULQIE scores) was lower in caregivers of children with ARPKD compared to contemporary caregivers of healthy children (who also bore special strains during the pandemic [11]), the subjective impact of the pandemic was rated lower by parents in ARPKD families than by contemporary control parents. Potentially, this reflects an increased focus on coping with the disease as a protective factor against the subjective feeling of being overwhelmed by contact restrictions and a global event out of their control.

The study was limited by the fact that no instruments are available to record symptoms of portal hypertension in children and the difficulty of quantifying hepatic morbidity in early disease, both because less reliable biomarkers are available and disease progression is less linear than decline in kidney function. Participant numbers were small compared to studies in healthy children, because of the very low incidence of ARPKD and the added difficulties of performing personal interviews during a time of fluctuating contact restrictions. Recruitment via treating physicians and self-help organisations may lead to a bias towards patients with better outcomes and/or more articulate families. Feasibility in terms of completion time was not assessed, but this has been reported for the PedsQL^®^ESRD and SDQ previously [22, 35, 49, 50].

In summary, ARPKD causes significantly impaired hrQOL, emotional and behavioural problems and caregiver burden, which were equal to, if not greater than, that of historical cohorts of children with all-cause kidney failure. More problems with peer interactions may be due to more stringent shielding of chronically ill children from social contacts during the COVID pandemic compared to healthy children. Additional developmental delay was a risk factor for both physical and mental well-being of the child, parental QOL and the disease’s impact on the family. For future clinical trials in ARPKD, better quantification of liver-related symptoms is needed.

## Supplementary Information

Below is the link to the electronic supplementary material.

ESM 1 Graphical abstract (PPTX 103 KB)

ESM 2 DOCX (147 KB)

## Data Availability

The dataset generated for and analyzed in the current study are not publicly available to protect patient confidentiality but are available in pseudonymized form from the corresponding author on reasonable request.

## Footnotes

1 Parents took part in a dual role both as proxies reporting on quality of life of their affected children, as well as caregivers who directly experience the burden of medical care for their child. To differentiate these aspects, the terms ‘proxy’ and ‘caregiver’ are used here.
