# Supplementary material for: Health-related quality of life, mental health and caregiver burden in children with autosomal recessive polycystic kidney disease
Source: Pediatr Nephrol. 2025 Sep 18;41(1):135–50. doi: 10.1007/s00467-025-06795-1 (PMC12686003; doi:10.1007/s00467-025-06795-1)
Supplement: Supplementary file 1 — Graphical abstract (PPTX 103 KB) [file 467_2025_6795_MOESM1_ESM.pptx]

## Slide 1
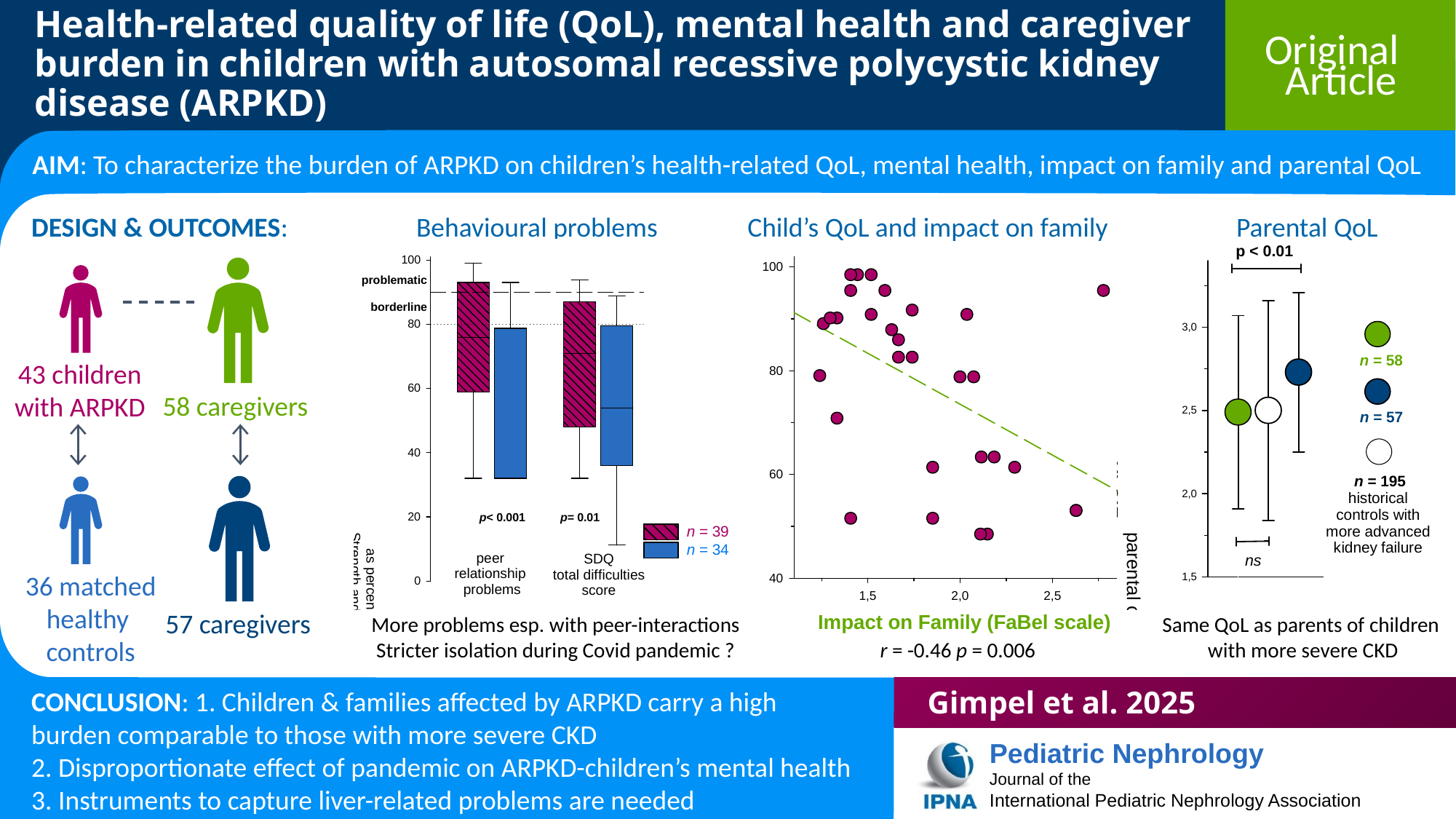

Health-related quality of life (QoL), mental health and caregiver burden in children with autosomal recessive polycystic kidney disease (ARPKD)
AIM: To characterize the burden of ARPKD on children’s health-related QoL, mental health, impact on family and parental QoL
DESIGN & OUTCOMES:
Behavioural problems
Child’s QoL and impact on family
Parental QoL
43 children with ARPKD
58 caregivers
36 matched healthy
controls
57 caregivers
More problems esp. with peer-interactions
Stricter isolation during Covid pandemic ?
Same QoL as parents of children
with more severe CKD
r = -0.46 p = 0.006
Gimpel et al. 2025
CONCLUSION: 1. Children & families affected by ARPKD carry a high burden comparable to those with more severe CKD
2. Disproportionate effect of pandemic on ARPKD-children’s mental health
3. Instruments to capture liver-related problems are needed
