# Supplementary material for: Health-related quality of life, mental health and caregiver burden in children with autosomal recessive polycystic kidney disease
Source: Pediatr Nephrol. 2025 Sep 18;41(1):135–50. doi: 10.1007/s00467-025-06795-1 (PMC12686003; doi:10.1007/s00467-025-06795-1)
Supplement: Supplementary file 2 — DOCX (147 KB) [file 467_2025_6795_MOESM2_ESM.docx]

**Health-related quality of life, mental health and caregiver burden in children with autosomal recessive polycystic kidney disease (ARPKD)**

Charlotte Gimpel^1,2^, Susanne Schaefer^1^, Franz Schaefer^1^

^1^ Division of Pediatric Nephrology, Center for Pediatrics and Adolescent Medicine, University Hospital Heidelberg, Heidelberg, Germany.

^2^ Praxis für Kinderkardiologie und Kindernephrologie, Medizinisches Versorgungszentrum des Klinikum Konstanz, Konstanz, Germany.

Corresponding author: PD Dr. med. Charlotte Gimpel: Charlotte.Gimpel@med.uni-hedielberg.de

**Supplementary Material**

# Content

[**Table S1** Number of completed questionnaires about children/adolescents 2](#_Toc193710590)

[**Table S2** Disease characteristics of children affected by ARPKD, for each questionnaire-subgroup 3](#_Toc193710591)

[**Table S3** Results of the PedsQL®ESRD 4](#_Toc193710592)

[**Table S4** Results of the strength and difficulties questionnaire (SDQ) 5](#_Toc193710593)

[**Table S5** Number of completed caregiver burden questionnaires 6](#_Toc193710594)

[**Table S6** Results of the Ulm inventory for parental quality of life (ULQIE) and Impact on Family score (FaBel scoring system) 7](#_Toc193710595)

[**Table S7** Correlation of the Ulm inventory for parental quality of life (ULQIE) and Impact on Family score (FaBel scoring system) 8](#_Toc193710596)

[**Table S8** Quality of life in parents with children affected by ARPKD compared to historical controls by treatment modality 9](#_Toc193710597)

[**Table S9** Impact on family subscales in families affected by ARPKD vs all-cause chronic kidney failure 10](#_Toc193710598)

[**Table S10** Child and parental quality of life indices of children with ARPKD by age at presentation 11](#_Toc193710599)

[**Figure S1** Strength and difficulties questionnaire (SDQ): proxy-reported subscores and total score in children with ARPKD who presented either before or up to 3 months post-natal (“perinatal”) or after 3 months of age (“later presentation”) 12](#_Toc193710600)

[**Figure S2** Strength and difficulties questionnaire (SDQ): proxy-reported subscores and total score in children with ARPKD with and without developmental delay secondary to disease complications 13](#_Toc193710601)

[**Figure S3** Subjective ranking of influence of pandemic on quality of life 14](#_Toc193710602)

## **Table S1** Number of completed questionnaires about children/adolescents

|  | | ARPKD | controls | *p* |
| --- | --- | --- | --- | --- |
| **Children**, total *n* | | 43 | 36 |  |
|  | |  |  |  |
| **Patient self-reports** | |  |  |  |
|  | Children with any self-report | 23/43 | 27/36 | ns |
| Type of self-report / number of eligible children: | | | | |
|  | PedsQL^®^ESRD | **21**/35 | **26**/26 | <0.001 |
|  | SDQ | **13**/15 | **12**/12 | ns |
|  |  |  |  |  |
| **Proxy-reports** | |  |  |  |
|  | Children with any proxy report | 42/43 | 33/36 | ns |
| Type of proxy-report* / number of eligible children: | | | | |
|  | PedsQL^®^ESRD | **39**/40 | **31**/34 | ns |
|  | PedsQL^®^infant | **3**/3 | **2**/2 | ns |
|  | SDQ | **39**/40 | **34**/34 | ns |
| Proxy-report completed by | | | | |
|  | Mother and Father | 19 (45%) | 4 (12%) | 0.004 |
|  | Mother | 18 (43%) | 27 (82%) |  |
|  | Father | 5 (12%) | 2 (6%) |  |
|  | Unknown | 1 (2%) | 3 (9%) |  |

ARPKD – autosomal dominant polycystic kidney disease.

PedsQL^®^ESRD – Pediatric quality of life inventory – end stage kidney disease module (for children from 5 years and proxies of children from 2 years of age).

SDQ – strength and difficulties questionnaire (for children from 11 years and proxies of children from 2 years of age).

PedsQL^®^infant – Pediatric quality of life inventory – infant module for proxies of children from 1 to 24 months of age.

*p* values are for chi-square test comparing proportions between affected families and controls.

ns – not significant
na – not applicable (questions could not be meaningfully posed to healthy controls)

* Where 2 parent-proxies answered for one child these are already combined

.

## **Table S2** Disease characteristics of children affected by ARPKD, for each questionnaire-subgroup

|  |  |  | *Patient’s hrQOL and mental health* | | | |  | *Caregiver burden* | |
| --- | --- | --- | --- | --- | --- | --- | --- | --- | --- |
|  | **All  ARPKD** |  | **PedsQL**®**ESRD**  **proxy** | **PedsQL**®**ESRD**  **self** | **SDQ**  **proxy** | **SDQ**  **self** |  | **ULQIE  (≥ 1 parent)** | **IFS**  **(≥ 1 parent)** |
| Children/ young persons [*n*] | 43 |  | 33 | 21 | 39 | 17 |  | 41 | 41 |
| Family units [*n*] | 39 |  | 30 | 21 | 35 | 16 |  | 37 | 37 |
| Age at interview [years] | 9.0 ± 4.8 |  | 10.2 ± 4.0 | 11.7 ± 4.6 | 9.3 ± 4.3 | 13.44 ± 3.5 |  | 8.4 ±4.2 | 8.9 ± 4.7 |
| Age at diagnosis [years] | 1.1 ± 2.7 |  | 1.3 ± 2.9 | 1.5 ± 3.4 | 1.3 ± 2.9 | 1.3 ± 3.7 |  | 0.9 ± 2.3 | 1.2 ± 2.8 |
| Female [*n*] | 20 (47%) |  | 17/33 (52%) | 12/21 (57%) | 19/39 (49%) | 10/17 (59%) |  | 19/41 (46%) | 20/41 (49%) |
| Height SDS [z-score] | -1.14 ± 1.35 |  | -1.04 ± 1.42 | -1.25 ± 1.39 | -1.11 ± 1.37 | -1.08 ± 1.61 |  | -1.12 ± 1.37 | -1.11 ± 1.37 |
| N^o^ of different drugs taken | 5.2 ± 4.2 |  | 4.5 ± 4.0 | 4.7 ± 3.7 | 4.7 ± 3.8 | 5.1 ± 4.2 |  | 5.3 ± 4.1 | 5.1 ± 4.2 |
| eGFR [ml/min*1.73m^2^] | 71 ± 47 |  | 74 ± 45 | 75 ± 47 | 76 ± 47 | 64 ± 41 |  | 74 ± 47 | 74 ± 47 |
| CKD stage 1-4 [*n*] | 24/42 (57%) |  | 21/32 (66%) | 12/20 (60%) | 24/38 (63%) | 8/17(47%) |  | 25/40 (63%) | 25/40 (63%) |
| CKD stage 5 [*n*] | 6/42 (14%) |  | 3/32(9%) | 1/20 (5%) | 4/38 (11%) | 1/17(6%) |  | 5/40 (13%) | 5/40 (13%) |
| Functioning graft after kidney, or liver & kidney transplant [*n*] | 11/42 (26%) |  | 8/32(25%) | 7/20 (35%) | 10/38 (26%) | 8/17(47%) |  | 10/40 (25%) | 10/40 (25%) |
| Perinatal presentation | 31/43 (72%) |  | 22/33 (67%) | 14/21 (67%) | 27/39 (69%) | 13/17 (76%) |  | 30/41 (73%) | 29/41 (71%) |
| Abdominal circumference SDS^#^ [z-score] | 1.5 ± 1.7 |  | 1.3 ± 1.5 | 0.76 ± 1.14 | 1.50 ± 1.73 | 0.86 ± 1.33 |  | 1.50 ± 1.68 | 1.50 ± 1.68 |
| Nephrectomy [*n*] | 12/43 (28%) |  | 7/33 (21%) | 5/21 (24%) | 9/39 (77%) | 5/17(29%) |  | 11/41 (27%) | 11/41 (27%) |
| Developmental delay [*n*] | 9/43 (21%) |  | 6/33 (18%) | 1/21 (5%) | 9/39 (23%) | 1/17(6%) |  | 9/41 (22%) | 9/41 (22%) |

hrQOL- health related quality of life

ARPKD – autosomal dominant polycystic kidney disease.

PedsQL^®^ESRD – Pediatric quality of life inventory – end stage kidney disease module (for children from 5 years and proxies of children from 2 years of age).

SDQ – strength and difficulties questionnaire (for children from 11 years and proxies of children from 2 years of age).

IFS – impact of family scale for families with an affected child.

ULQIE – Ulm quality of life inventory for parents of a child with chronic illness.

^#^ Only patients who had not undergone nephrectomy

## **Table S3** Results of the PedsQL®ESRD

|  |  | ARPKD | | | |  | Control | | | |  | *p* |
| --- | --- | --- | --- | --- | --- | --- | --- | --- | --- | --- | --- | --- |
| Informant | **PedsQL** **ESRD Subscale** | **n** | **Mean** | **SD** | **α** |  | **n** | **Mean** | **SD** | **α** |  |  |
| Proxy | Fatigue | 38 | 74.3 | 17.3 | 0.85 |  | 30 | 79.9 | 17.3 | 0.88 |  | 0.05 |
|  | Renal symptoms | 38 | 83.1 | 13.4 | 0.46 |  | 30 | 89.9 | 7.4 | 0.09 |  | 0.02 |
|  | Treatment | 36 | 72.3 | 18.8 | 0.39 |  | - | - | - |  |  | - |
|  | - Food and drink only* | 33 | 70.1 | 25.7 | 0.56 |  | 29 | 81.7 | 20.6 | 0.41 |  | 0.03 |
|  | Social interaction | 33 | 85.9 | 18.5 | 0.80 |  | - | - | - | - |  | - |
|  | Worry | 33 | 77.8 | 15.3 | 0.84 |  | - | - | - | - |  | - |
|  | Appearance | 32 | 87.1 | 15.5 | 0.45 |  | - | - | - | - |  | - |
|  | Communication | 34 | 68.6 | 27.4 | 0.94 |  | - | - | - | - |  | - |
|  | **Total** | **33** | **78.1** | **10.8** | **0.88** |  | - | - | - | - |  | - |
| Self | Fatigue | 22 | 79.8 | 20.5 | 0.79 |  | 26 | 81.7 | 14.6 | 0.62 |  | ns |
|  | Renal symptoms | 22 | 82.1 | 14.5 | 0.38 |  | 26 | 86.3 | 13.5 | 0.52 |  | ns |
|  | Treatment | 22 | 82.6 | 16.3 | 0.33 |  | - | - | - | - |  | - |
|  | - Food and drink only* | 29 | 81.7 | 20.6 | 0.17 |  | 26 | 78.4 | 19.2 | -0.07 |  | ns |
|  | Social interaction | 21 | 82.1 | 26.9 | 0.84 |  | - | - | - | - |  | - |
|  | Worry | 21 | 76.0 | 21.6 | 0.88 |  | - | - | - | - |  | - |
|  | Appearance | 20 | 85.0 | 21.6 | 0.45 |  | - | - | - | - |  | - |
|  | Communication | 21 | 75.8 | 21.7 | 0.77 |  | - | - | - | - |  | - |
|  | **Total** | **21** | **79.2** | **16.6** | **0.95** |  | - | - | - | - |  | - |

α standardized Cronbach’s alpha

SD standard deviation

*P* values are for one-sided Wilcoxon test comparing scores of ARPKD vs control groups

- not applicable (questions could not be meaningfully posed to healthy controls)

* “food and drink only” is not an official subscale of the PedsQL®ESRD, but a summary of 2 treatment-related questions that can be meaningfully posed to (proxies of) healthy children (“In the last month, how much of a problem has your child had with …” (1) “drinking the amount of fluid he/she is supposed to.” and (2) “… getting upset when he/she cannot eat foods that he/she wants to eat.”)

## **Table S4** Results of the strength and difficulties questionnaire (SDQ)

|  |  | ARPKD | | | |  | Control | | | |  | *p* |
| --- | --- | --- | --- | --- | --- | --- | --- | --- | --- | --- | --- | --- |
| Informant | **SDQ Subscale** | **n** | **Mean** | **SD** | **α** |  | **n** | **Mean** | **SD** | **α** |  |  |
| Proxy | Emotional symptoms | 39 | 2.59 | 2.12 | 0.68 |  | 34 | 2.18 | 2.32 | 0.76 |  | ns |
|  | Conduct problems | 39 | 2.10 | 1.40 | 0.57 |  | 34 | 2.09 | 1.42 | 0.46 |  | ns |
|  | Hyperactivity/ inattention | 39 | 4.40 | 2.67 | 0.76 |  | 34 | 3.00 | 2.44 | 0.82 |  | 0.01 |
|  | Peer relationship problems | 39 | 2.53 | 2.02 | 0.57 |  | 34 | 1.18 | 1.64 | 0.63 |  | <0.001 |
|  | Prosocial behaviour | 39 | 7.73 | 1.84 | 0.75 |  | 34 | 7.85 | 1.73 | 0.71 |  | ns |
|  | **Total difficulties score** | **39** | **11.56** | **5.72** | **0.79** |  | **34** | **8.44** | **5.49** | **0.81** |  | **0.01** |
|  | Impact of difficulties | 38 | 1.30 | 1.81 | 0.76 |  | 34 | 0.62 | 1.16 | 0.27 |  | 0.02 |
| Self | Emotional symptoms | 17 | 2.24 | 2.25 | 0.76 |  | 12 | 2.75 | 3.28 | 0.92 |  | ns |
|  | Conduct problems | 17 | 2.00 | 1.00 | -0.39 |  | 12 | 1.58 | 1.68 | 0.61 |  | ns |
|  | Hyperactivity/ inattention | 17 | 3.94 | 1.98 | 0.47 |  | 12 | 3.33 | 2.74 | 0.87 |  | ns |
|  | Peer relationship problems | 17 | 2.41 | 1.54 | 0.37 |  | 12 | 1.33 | 0.78 | -0.66 |  | 0.02 |
|  | Prosocial behaviour | 17 | 8.18 | 1.85 | 0.72 |  | 12 | 8.50 | 1.24 | 0.32 |  | ns |
|  | **Total difficulties score** | **17** | **10.59** | **5.23** | **0.69** |  | **12** | **9.00** | **6.58** | **0.87** |  | **ns** |
|  | Impact of difficulties | 17 | 1.06 | 1.34 | 0.64 |  | 12 | 0.92 | 1.51 | 0.32 |  | ns |

α standardized Cronbach’s alpha

SD standard deviation

*P* values are for one-sided Wilcoxon test comparing scores of ARPKD vs control groups

## **Table S5** Number of completed caregiver burden questionnaires

|  |  | ARPKD | controls | *p* |
| --- | --- | --- | --- | --- |
| **Family units** | | 39 | 35 |  |
|  | Any parental report | 38 (97%) | 35 (100%) | ns |
|  | Mothers – any report | 36 (92%) | 34 (97%) | ns |
|  | Fathers – any report | 22 (56%) | 23 (66%) | ns |
|  |  |  |  |  |
| **Impact on family scale (FaBel)** | | | | |
|  | Mothers | 35 (90%) | na | na |
|  | Fathers | 22 (56%) | na | na |
| **Parental quality of life (ULQIE)** | | | | |
|  | Mothers | 36 (92%) | 34 (97%) | ns |
|  | Fathers | 22 (56%) | 23 (66%) | ns |

ARPKD – autosomal dominant polycystic kidney disease.

FaBel – impact of family scale for families with an affected child.

ULQIE – Ulm quality of life inventory for parents of a child with chronic illness (parents of healthy children were also eligible).

*p* values are for chi-square test comparing proportions between affected and control families

## **Table S6** Results of the Ulm inventory for parental quality of life (ULQIE) and Impact on Family score (FaBel scoring system)

|  | | | ARPKD | | | |  | Control | | | |  |  |
| --- | --- | --- | --- | --- | --- | --- | --- | --- | --- | --- | --- | --- | --- |
|  | | | **n** | **Mean** | **SD** | **α** |  | **n** | **Mean** | **SD** | **α** |  | **p** |
| ULQIE total score | | | **58** | **2.49** | **0.58** | **0.94** |  | **57** | **2.73** | **0.48** | **0.93** |  | **<0.001** |
|  | | Physical and daily functioning | 58 | 2.47 | 0.66 | 0.82 |  | 57 | 2.83 | 0.64 | 0.86 |  | 0.07 |
|  | | Satisfaction with family support | 58 | 3.21 | 0.67 | 0.80 |  | 57 | 3.12 | 0.55 | 0.86 |  | 0.06 |
|  | | Emotional strain | 58 | 2.28 | 0.87 | 0.76 |  | 57 | 2.54 | 0.70 | 0.69 |  | 0.02 |
|  | | Self-development | 58 | 1.60 | 0.94 | 0.85 |  | 57 | 1.90 | 0.85 | 0.83 |  | <0.001 |
|  | | General well-being | 58 | 2.59 | 0.73 | 0.67 |  | 57 | 3.02 | 0.63 | 0.75 |  | <0.01 |
|  | | |  |  |  |  |  |  |  |  |  |  |  |
| FaBel 27 item total score | | | **57** | **2.01** | **0.52** | **0.90** |  |  |  |  |  |  |  |
| FaBel 11 item total score | | | **57** | **2.10** | **0.83** | **0.94** |  |  |  |  |  |  |  |
|  | Daily psychosocial strains | | 57 | 2.02 | 0.70 | 0.92 |  |  |  |  |  |  |  |
|  | Siblings′ psychosocial strains | | 39 | 1.68 | 0.54 | 0.70 |  |  |  |  |  |  |  |
|  | Financial burden | | 57 | 1.79 | 0.67 | 0.75 |  |  |  |  |  |  |  |
|  | Emotional strains and worries concerning the future | | 57 | 2.38 | 0.54 | 0.42 |  |  |  |  |  |  |  |
|  | Problems with coping | | 57 | 1.64 | 0.64 | 0.62 |  |  |  |  |  |  |  |

## **Table S7** Correlation of the Ulm inventory for parental quality of life (ULQIE) and Impact on Family score (FaBel scoring system)

|  |  | ULQIE | | | | | | |
| --- | --- | --- | --- | --- | --- | --- | --- | --- |
| FaBel |  | **Total  score** |  | Physical and daily functioning | Satisfaction with family support | Emotional strain | Self-development | General well-being |
| Total score  (27 item) | *r* | **-0.72** |  | **-0.6** | **-0.3** | **-0.74** | **-0.58** | **-0.66** |
|  | *p* | **<.0001** |  | **<.0001** | **0.02** | **<.0001** | **<.0001** | **<.0001** |
|  |  |  |  |  |  |  |  |  |
| Daily psychosocial strains | *r* | **-0.69** |  | **-0.59** | -0.24 | **-0.72** | **-0.58** | **-0.64** |
|  | *p* | **<.0001** |  | **<.0001** | 0.08 | **<.0001** | **<.0001** | **<.0001** |
| Siblings′ psychosocial strains (n=39) | *r* | **-0.7** |  | **-0.61** | -0.32 | **-0.35** | -0.29 | **-0.67** |
|  | *p* | **<.0001** |  | **<.0001** | 0.05 | **0.03** | 0.08 | **<.0001** |
| Financial  burden | *r* | **-0.62** |  | **-0.53** | **-0.32** | **-0.58** | **-0.47** | **-0.63** |
|  | *p* | **<.0001** |  | **<.0001** | **0.01** | **<.0001** | **0.0003** | **<.0001** |
| Emotional strains & worries about future | *r* | **-0.28** |  | -0.19 | -0.05 | **-0.39** | **-0.27** | -0.21 |
|  | *p* | **0.03** |  | 0.17 | ns | **0.003** | **0.045** | 0.13 |
| Problems with coping | *r* | -0.25 |  | -0.18 | **-0.36** | -0.15 | -0.08 | -0.13 |
|  | *p* | 0.056 |  | 0.17 | **0.006** | ns | ns | ns |

*r*: Pearson’s correlation coefficient

## **Table S8** Quality of life in parents with children affected by ARPKD compared to historical controls by treatment modality

|  | Parents of children  with ARPKD | | |  | Parents of children with advanced CKF* | |
| --- | --- | --- | --- | --- | --- | --- |
|  | mean | SD | Range |  | mean | SD |
| *CKD stage G1-4* | *n=26* | | |  | *n=30* | |
| ULQIE-total score | 2.79 | 0.51 | 1.7-3.9 |  | 2.65 | n.a. |
| Physical and daily functioning | 2.75 | 0.61 | 1.1-3.9 |  | 2.67 | n.a. |
| Satisfaction with family support | 3.35 | 0.60 | 1.5-4.0 |  | 3.01 | n.a. |
| Emotional strain | 2.74 | 0.74 | 0.8-4.0 |  | 2.50 | n.a. |
| Self-development | 2.04 | 1.06 | 0.5-4.0 |  | 2.07 | n.a. |
| General well-being | 2.97 | 0.57 | 1.8-3.8 |  | 2.90 | n.a. |
|  |  |  |  |  |  |  |
| *Dialysis* | *n=8* | | |  | *n=32* | |
| ULQIE-total score | 2.14 | 0.56 | 1.0-2.8 |  | 2.33 | n.a. |
| Physical and daily functioning | 1.96 | 0.67 | 0.7-2.9 |  | 2.29 | n.a. |
| Satisfaction with family support | 3.13 | 0.53 | 2.0-4.0 |  | 3.08 | n.a. |
| Emotional strain | 1.53 | 0.93 | 0.3-3.3 |  | 1.91 | n.a. |
| Self-development | 1.28 | 0.88 | 0.3-2.8 |  | 1.60 | n.a. |
| General well-being | 2.16 | 0.88 | 0.8-3.3 |  | 2.26 | n.a. |
|  |  |  |  |  |  |  |
| *Functioning kidney graft* | *n=16* | | |  | *n=121* | |
| ULQIE-total score | 2.20 | 0.64 | 1.0-3.5 |  | 2.52 | n.a. |
| Physical and daily functioning | 2.27 | 0.67 | 1.3-3.7 |  | 2.53 | n.a. |
| Satisfaction with family support | 2.96 | 0.88 | 1.5-4.0 |  | 2.92 | n.a. |
| Emotional strain | 1.89 | 0.68 | 1.0-3.3 |  | 2.38 | n.a. |
| Self-development | 1.23 | 0.96 | 0.0-3.8 |  | 1.91 | n.a. |
| General well-being | 2.14 | 0.72 | 1.0-3.3 |  | 2.64 | n.a. |

* Parents of children with advanced CKF are historical controls from Wiedebusch et al. “Health-related quality of life, psychosocial strains, and coping in parents of children with chronic renal failure” Ped Nephrology 2010, 25:1477-1485.

ARPKD – autosomal dominant polycystic kidney disease.

CKF – chronic kidney failure

ULQIE – Ulm quality of life inventory for parents of a child with chronic illness (parents of healthy children were also eligible).

n.a. not available

## **Table S9** Impact on family subscales in families affected by ARPKD vs all-cause chronic kidney failure

|  | Families affected by ARPKD | Families affected by all-cause CKF* | *p* value |
| --- | --- | --- | --- |
| Parents, *n* | 58 | 195 |  |
|  |  |  |  |
| FaBel subscores: | mean ± SD | mean ± SD |  |
| Daily psychosocial strains | 2.02 ± 0.70 | 2.31 ± 0.58 | 0.004 |
| Siblings′ psychosocial strains | 1.68 ± 0.54 | 1.97 ± 0.62 | <0.001 |
| Financial burden | 1.79 ± 0.67 | 2.19 ± 0.78 | <0.001 |
| Emotional strains and worries   concerning the future | 2.38 ± 0.54 | 2.46 ± 0.58 | ns |
| Problems with coping | 1.64 ± 0.64 | 1.76 ± 0.63 | ns |
|  |  |  |  |
| Duration of illness  (<1year/1-5years/>5years/unknown) | 6%/24%/70%/0% | 7%/21%/67%/5% | ns |
| Treatment modality  (CKD stage G1-4/dialysis/TPL/unknown) | 62%/10%/25%/3% | 15%/16%/62%/6% | <0.001 |

German FaBel scoring (range from 1 to 4, higher numbers indicate higher impact)

ARPKD: autosomal recessive polycystic kidney disease

CKF: chronic kidney failure

TPL: after kidney transplantation

* Families affected by all-cause CKF are historical controls from Wiedebusch et al. “Health-related quality of life, psychosocial strains, and coping in parents of children with chronic renal failure” Ped Nephrology 2010, 25:1477-1485.

*P* values comparing means are for Welch’s *t-*test of ARPKD vs all-cause CKF (which might overestimate significance as it assumes normal distribution)

*P* values comparing proportions are for chi square test

## **Table S10** Child and parental quality of life indices of children with ARPKD by age at presentation

|  | Perinatal presentation | | | |  | Presentation > 3 months | | | | |  | |  |
| --- | --- | --- | --- | --- | --- | --- | --- | --- | --- | --- | --- | --- | --- |
| Variable | n | mean | SD | range |  | n | mean | SD | range |  | | ***p*** | |
| PedsQL®ESRD Total, proxy-rated | 22 | 78.0 | 10.4 | (63-95) |  | 11 | 78.3 | 12.1 | (61-92) |  | | ns | |
| PedsQL®ESRD Total,  self-reported | 14 | 79.8 | 16.7 | (48-98) |  | 7 | 77.9 | 17.8 | (52-98) |  | | ns | |
|  |  |  |  |  |  |  |  |  |  |  | |  | |
| SDQ Total score,  proxy-rated | 27 | 12.9 | 5.0 | (4-22) |  | 12 | 8.58 | 6.3 | (3-24) |  | | **0.01** | |
| SDQ Total score,  self-reported | 13 | 11.0 | 5.8 | (5-23) |  | 4 | 9.25 | 2.8 | (7-13) |  | | ns | |
|  |  |  |  |  |  |  |  |  |  |  | |  | |
| ULQIE Total Score | 30 | 2.39 | 0.53 | (1.03-3.55) |  | 11 | 2.93 | 0.56 | (1.91-3.83) |  | | **0.005** | |
| FaBel Total Score | 30 | 2.12 | 0.54 | (1.24-3.28) |  | 11 | 1.65 | 0.27 | (1.26-2.19) |  | | **0.008** | |
|  |  |  |  |  |  |  |  |  |  |  | |  | |
| Age at interview [years] | 31 | 8.7 | 4.9 | (0.5-19.3) |  | 12 | 9.7 | 4.8 | (3.9-21.4) |  | | ns | |
| Age at diagnosis [years] | 31 | -0.10 | 0.14 | (-0.47-0.24) |  | 12 | 4.30 | 3.65 | (0.5-11) |  | | **<0.0001** | |
| eGFR [ml/min*1.73 m^2^] | 24 | 58 | 41 | (4-140) |  | 9 | 108 | 44 | (5-140) |  | | **0.004** | |
| Height SDS [z-score] | 30 | -1.27 | 1.52 | (-4.77-1.67) |  | 12 | -0.80 | 0.73 | (-1.7-0.52) |  | | ns | |
| Nr of different medications taken | 29 | 6.4 | 4.3 | (0-16) |  | 12 | 2.4 | 1.9 | (0-7) |  | | **0.003** | |
|  |  |  |  |  |  |  |  |  |  |  | |  | |
| Treatment modality CKD stage G1-4 |  | 14 | of 31 | (=45%) |  |  | 11 | of 12 | (92%) |  | | **0.02** | |
| Developmental delay |  | 8 | of 31 | (=26%) |  |  | 1 | of 12 | (8%) |  | | ns | |

PedsQL®ESRD – Pediatric quality of life inventory – end stage kidney disease module (for children from 5 years and proxies of children from 2 years of age).

SDQ – strength and difficulties questionnaire (for children from 11 years and proxies of children from 2 years of age)

ULQIE – Ulm quality of life inventory for parents of a child with chronic illness (if both parents answered mean was taken)

FaBel – impact of family scale for families with an affected child (if both parents answered mean was taken)

ns – not significant

*p* values are for the non-parametric one-sided Wilcoxon test

## **Figure S1** Strength and difficulties questionnaire (SDQ): proxy-reported subscores and total score in children with ARPKD who presented either before or up to 3 months post-natal (“perinatal”) or after 3 months of age (“later presentation”)

Box: 25^th^, 50^th^ and 75^th^ percentile. Whiskers: 5^th^ and 95^th^ percentile.

*P* values are for one-sided Wilcoxon test of SDQ raw scores in perinatal vs later presentation group

## **Figure S2** Strength and difficulties questionnaire (SDQ): proxy-reported subscores and total score in children with ARPKD with and without developmental delay secondary to disease complications

## **Figure S3** Subjective ranking of influence of pandemic on quality of life

* participants answered on 5-point Lickert scale. If more than one person answered (e.g. mother and father) mean in shown.
